# A Phase II Study of Arginine Deiminase (ADI-PEG20) in Relapsed/Refractory or Poor-Risk Acute Myeloid Leukemia Patients

**DOI:** 10.1038/s41598-017-10542-4

**Published:** 2017-09-12

**Authors:** Hui-Jen Tsai, Shih Sheng Jiang, Wen-Chun Hung, Gautam Borthakur, Sheng-Fung Lin, Naveen Pemmaraju, Elias Jabbour, John S. Bomalaski, Ya-Ping Chen, Hui-Hua Hsiao, Ming-Chung Wang, Ching-Yuan Kuo, Hung Chang, Su-Peng Yeh, Jorge Cortes, Li-Tzong Chen, Tsai-Yun Chen

**Affiliations:** 1 National Institute of Cancer Research, National Health Research Institutes, Tainan, Taiwan; 2 Division of Hematology/Oncology, Department of Internal Medicine, National Cheng Kung University Hospital, Tainan, Taiwan; 3 Department of Internal Medicine, Kaohsiung Medical University Hospital, and Kaohsiung Medical University, Kaohsiung, Taiwan; 4 Department of Leukemia, Division of Cancer Medicine, University of Texas M.D. Anderson Cancer Center, Houston, TX USA; 5 Polaris Pharmaceuticals, Inc., Polaris Group, San Diego, CA USA; 6 Department of Internal Medicine, Kaohsiung Chang Gung Memorial Hospital, Kaohsiung, Taiwan; 7 Department of Internal Medicine, Linkou Chang Gung Memorial Hospital, Linkou, Taiwan; 8 Department of Internal Medicine, China Medical University Hospital, Taichung, Taiwan; 9 Institute of Molecular Medicine, National Cheng Kung University, Tainan, Taiwan

## Abstract

Exogenous arginine is required for growth in some argininosuccinate synthetase (ASS)-deficient cancers. Arginine deiminase (ADI) inhibits growth in various ASS-deficient cancers by depleting arginine. The efficacy of pegylated ADI (ADI-PEG20) in relapsed/refractory/poor-risk acute myeloid leukemia (AML) was evaluated in 43 patients in a prospective, phase II trial (NCT01910012 (10/07/2013), https://clinicaltrials.gov/ct2/show/NCT01910012?term = ADI-PEG20&rank = 12). Despite almost all pre-treatment tumor samples showing ASS deficiency, the best response among 21 evaluable patients was complete response (CR) in 2 (9.5%) and stable disease in 7 (33.3%), yielding a disease control rate (DCR) of 42.9%. The response durations of the two patients with CR were 7.5 and 8.8 months. DCR was correlated with a median of 8 weeks of arginine depletion to ≤10 μM. Using whole transcriptome sequencing, we compared gene expression profiling of pre- and post-treatment bone marrow samples of the two responders and three non-responders. The expression levels of some markers for AML subtypes and c-MYC regulated genes were considered potential predictors of response to ADI-PEG20. These results suggest that ASS deficiency is a prerequisite but not a sufficient condition for response to ADI-PEG20 monotherapy in AML. Predictive biomarkers and mechanistic explorations will be critical for identifying appropriate patients for future AML trials of ADI-PEG20.

## Introduction

Acute myeloid leukemia (AML) is a heterogeneous clonal disorder characterized by an increase in the number of immature myeloid cells and an arrest of their maturation in the bone marrow (BM)^1^. Approximately 50% of AML cases are diagnosed at an age older than 60 years^2^. Intensive chemotherapy induces a complete remission (CR) rate of >70% in younger patients but only approximately 50% in elderly patients^3, 4^. More than half of CR patients will relapse within 2 years^3^. Comprehensive studies of cytogenetic and molecular aberrations have provided important insights into the pathogenesis, diagnosis, prognosis, and treatment strategy for AML in recent decades^5^. Many novel agents, such as FLT3, MEK, and aurora kinase inhibitors, have been investigated as single agents for AML treatment^6–8^, but the results have been unsatisfactory.

Arginine, a semi-essential amino acid in humans, is critical for the growth of certain human cancers. In addition to protein synthesis, arginine is involved in diverse aspects of tumor metabolism^9^. Deficiency in argininosuccinate synthetase (ASS), the rate-limiting enzyme for endogenous arginine production in the urea cycle, has been found in various cancer tissues^9^. Arginine deprivation, either by restriction of arginine supplementation in tissue culture media or by treatment with arginine deiminase (ADI), a mycoplasma enzyme, has been shown to kill tumor cells but not normal cells^10, 11^. The role of ASS is clear, and a difference in intrinsic arginine deprivation between plasma and malignant cells is apparent. In preclinical studies, pegylated arginine deiminase (ADI-PEG20), which can rapidly convert arginine into citrulline, was shown to exert *in vitro* and *in vivo* anti-proliferative effects on ASS-deficient cancers, such as hepatocellular carcinoma, melanoma, prostate cancer, and lymphoma^12–14^. The efficacy of ADI-PEG20 in treating various solid tumors is currently being studied in clinical trials^15–17^.

Absence of ASS expression has been noted in 87% (46/53) of BM biopsy samples from AML cases^18^, and ADI-PEG20 was shown to kill leukemic cells *in vitro* and *in vivo* ^19^. Therefore, a phase II trial evaluating the therapeutic efficacy of ADI-PEG20 monotherapy in relapsed/refractory and/or poor-risk AML patients was conducted.

## Results

### Patient characteristics and treatment delivered

A total of 43 patients were enrolled from Oct 2013 to Oct 2015 in Taiwan and the US. Twenty-two patients were not evaluable because they received only one dose of ADI-PEG20, died early due to disease-related conditions, or did not have BM aspiration performed after 2 cycles of ADI-PEG20. Twenty-one patients were evaluable for tumor response. The baseline characteristics of all of the intention-to-treat (ITT) patients are listed in Table 1. The median age of the evaluable patients was 67.3 years (range, 28.3 to 85 years). Only 5 patients had an Eastern Cooperative Oncology Group (ECOG) performance of 2, and the other 38 patients had an ECOG performance of 0 or 1. Nine of the 43patients were chemo-naïve, and their ages were greater than 65 years. Nineteen of 43 patients had received more than 3 types of chemotherapy, and 3 of these 19 patients had undergone previous allogeneic stem cell transplantation. The median initial WBC count was 3700/μl (ranging from 400to 121,400/μl), with initial WBC counts greater than 10000/μl in 11 patients. Among the 43 ITT patients, the median time of treatment was 8 weeks (range, 1~41 weeks). Four patients received ≥6 cycles of ADI-PEG20, one of whom is still receiving treatment. One patient stopped receiving ADI-PEG20 at cycle 5 due to the occurrence of suspected anaphylactic shock. The other patients received 1~12 weeks of ADI-PEG20, and the study drug was withheld due to early death, progressive disease (PD) or lack of clinical benefit.

**Table 1:** Patient demographics and baseline characteristics.

Characteristics	Patients	
	N	%
Age (y/o)		
Median 67.3		
Range 28.3~85		
≤60	14	33
60~70	11	26
70~80	14	33
>80	4	9
Sex		
Male	21	49
Female	22	51
Prior chemotherapy		
Yes	34	79
1–2 lines	15	35
≥3 lines (SCT*)	19 (3)	44(7)
No	9	21
ECOG		
0	12	28
1	26	60
2	5	12
Initial WBC count(/μl)		
Median 3700		
Range 400~121,400		
>10000/μl	11	26
≤10000/μl	32	74
Duration of Treatment (weeks)		
Median 8		
Range 1~41		

^*^SCT, stem cell transplantation.

### Efficacy

**Figure 1:**
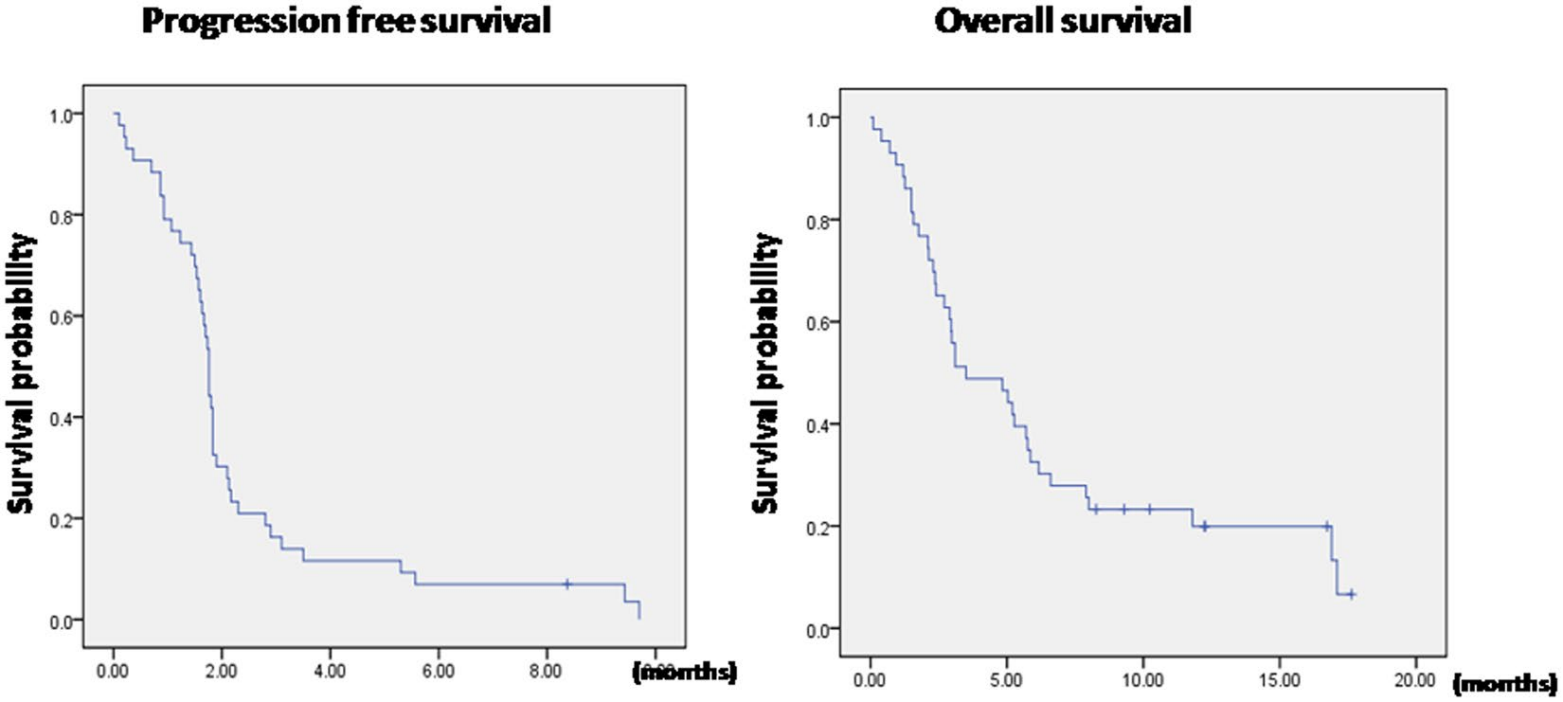
Progression-free survival and overall survival of all intention-to-treat patients.

Among the 43 ITT patients, the CR rate was 4.7% (95% C.I., 0~11.2%). The stable disease rate was 16.3% (95% C.I., 4.8%~27.8%). The median progression-free survival (PFS) and overall survival (OS) were 1.8 months (95% C.I., 1.7–1.8 months) and 3.5 months (95% C.I., 0.9–6.1 months), respectively. The PFS and OS of the ITT patients are shown in Fig. 1. For the 21 evaluable patients, the best response to ADI-PEG20 was observed in 2 patients, who achieved CR; 7 patients achieved stable disease (SD), whereas the other 12 patients experienced PD. The response rate was 9.5% (95% C.I., 1.2%~30.4%), and the disease control rate was 42.9% (95% C.I., 21.8%~66.0%). The median duration of ADI-PEG20 treatment among the patients with CR and SD was 17 weeks (range, 8~41 weeks). One patient with SD received 41weeks of ADI-PEG20 and is still receiving treatment. This patient had hematologic improvement in trilineage hematopoiesis after 3 cycles of ADI-PEG20 and became transfusion-independent. Another two SD patients had hematologic improvement: one patient gained improvements in hemoglobin level and the other patient gained improvements in neutrophil count. The patient characteristics, treatment duration, best response, duration of arginine deprivation, and survival are listed in Table 2.The median PFS and OS were 1.8 months (95% C.I., 1.8–2.8 months) and 7.9 months (95% C.I., 3.5–16.9 months), respectively. For the 2 patients with CR, the durations of response were 7.5 and 8.8 months, respectively. For the 7 patients with SD, the median time to disease progression was 3.5 months (range, 1.8~8.4 months). The baseline marrow blast, FAB subtypes and cytogenetic profile of the 21 evaluable patients were classified into CR + SD and PD and are listed in Supplementary Table 1. There was no significant difference in the baseline marrow blast between the two groups.

**Table 2:** Characteristics and efficacy of the evaluable patients.

Patient No.	Age	Sex^#^	Duration of treatment (weeks)	Duration of arginine deprivation (weeks)	Response*	PFS (months)	OS (months)	Survival
1	78.8	M	24	16	CR	9.43	17.1	death
2	69.6	F	17	5	CR	9.70	16.9	death
3	75.1	F	8	8	PD	1.76	11.8	death
4	71.2	M	8	5	PD	1.83	5.86	death
5	80.8	M	8	4	PD	1.76	2.7	death
6	65.4	M	41	5	SD	8.37	9.3	alive
7	48.4	F	8	9	PD	1.83	2.97	death
8	54.5	M	8	2	PD	1.83	7.9	death
9	65.6	M	10	8	SD	1.76	2.37	death
10	36.9	F	8	8	PD	1.80	2.4	death
11	83.4	F	8	8	PD	1.70	4.83	death
12	70	M	24	5	SD	5.30	5.27	death
13	74.2	F	8	8	PD	1.83	8	death
14	50	M	24	25	SD	5.57	12.23	alive
15	31.2	M	8	8	PD	1.76	5.2	death
16	58.1	M	9	8	SD	2.1	2.1	death
17	76.9	M	12	13	SD	2.8	16.73	alive
18	53.3	F	8	8	SD	3.5	3.5	death
19	80	M	8	8	PD	1.6	12.27	alive
20	85	F	8	5	PD	1.90	10.23	alive
21	72	F	8	NA**	PD	1.67	8.27	alive

^#^M: Male, F: Female.

^*^CR: complete remission, SD: stable disease, PD: progressive disease.

**NA: not available.

### Toxicity and pharmacodynamics

All of the ITT patients were evaluated for toxicities as listed in Table 3. Overall, the toxicity was minimal and tolerated. The most common treatment-related adverse events were grade 1/2 skin rash (9.3%), grade 1/2 hyperuricemia (7%), and grade 3/4 leukopenia (7%). There were 2 patients who had grade 3/4 neutropenic fever (4.7%), and 2 patients had grade 3/4 anemia (4.7%). One patient had grade 4 tumor lysis syndrome and an infection and died; the death was attributed to both grade 4 events, consistent with AML and possibly ADI-PEG20. Due to the development of tumor lysis syndrome in this patient, prophylactic use of allopurinol was subsequently prescribed for all of the enrolled patients. Another patient had grade 4 anaphylactic shock and recovered without sequelae.

**Table 3:** Adverse events related to ADI-PEG20.

Grade	1/2		3/4	
Total N = 43	N	%	N	%
Skin rash	4	9.3	0	0
Itching	1	2.3	0	0
Edema	1	2.3	0	0
Pain	2	4.7	0	0
Nausea	1	2.3	0	0
Vomiting	1	2.3	0	0
Fatigue/Weakness	1	2.3	1	2.3
Insomnia	1	2.3	0	0
Hyperuricemia	3	7	0	0
Tumor lysis syndrome	0	0	1	2.3
Infection	1	2.3	1	2.3
Neutropenic fever	0	0	2	4.7
Anaphylactic shock	0	0	1	2.3
Hyponatremia	0	0	1	2.3
Liver function impairment	1	2.3	1	2.3
Leukopenia	0	0	3	7.0
Anemia	0	0	2	4.7

**Figure 2:**
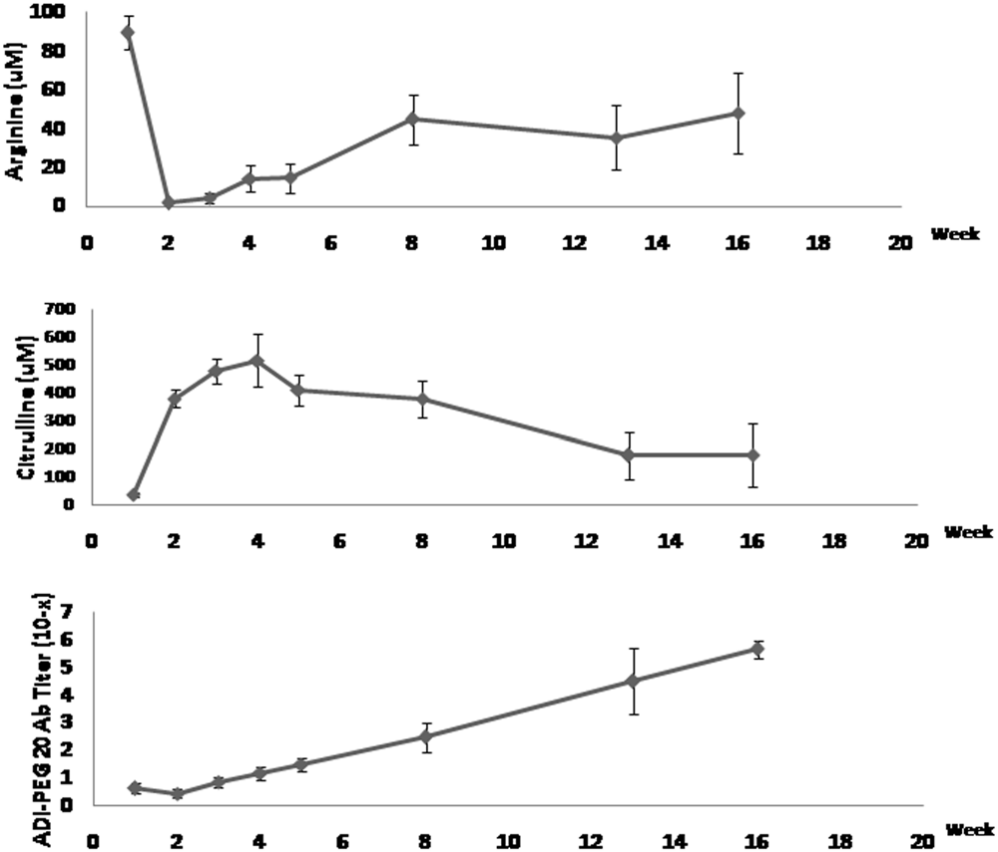
The mean circulating arginine and citrulline levels and the titers of anti-ADI-PEG20 antibodies in the intention-to treat patients during the ADI-PEG20 treatment.

The circulating arginine level was markedly decreased, accompanied by an increase in circulating citrulline levels after the first dose of ADI-PEG20 for all of the ITT patients, as shown in Fig. 2. The mean arginine level at baseline was 89 ± 8.7 μM, and the mean arginine level remained less than 10 μM for 2 weeks after starting ADI-PEG20 therapy and increased gradually. Pharmacodynamics and immunogenicity were assessed on day 8 after the prior ADI-PEG20 dose. The anti-ADI-PEG20 antibody levels increased beginning in the third week after ADI-PEG20 treatment, as shown in Fig. 2. For the 9 patients who had CR or SD after ADI-PEG20 treatment, the arginine level remained nearly undetectable for a median of 8 weeks (range 5–25) and gradually increased thereafter. One CR patient and one SD patient had low arginine levels (less than 10 μM) for 16 and 25 weeks, respectively, after ADI-PEG20 treatment. The case with CR developed anti-ADI-PEG20 antibody at a titer of 10^−2^ beginning in the second week after ADI-PEG20 and had the highest titer of the antibody (10^−6^) detected at the twentieth week after ADI-PEG 20 treatment. Another patient achieved CR after 1 cycle of ADI-PEG20, and the arginine level of this patient remained low for 5 weeks. This patient developed anti-ADI-PEG20 antibody at a titer of 10^−2^ beginning in the fourth week after ADI-PEG20 and had the highest titer of antibody (10^−6^) detected at the eighth week after ADI-PEG 20 treatment. ASS levels were determined from pre-enrollment samples in 17 of 21 patients. All 17 patients were ASS-deficient, with a median of 95% deficiency (range 81–100%). BM ASS level was determined at the time of disease progression in 10 of 12 patients with PD as the best response, and ASS pre-enrollment was available for 8 of the 10 patients. The ASS expression in the BM of these AML patients was not significantly increased after ADI-PEG20 treatment.

### Clinical details of responders

**Figure 3:**
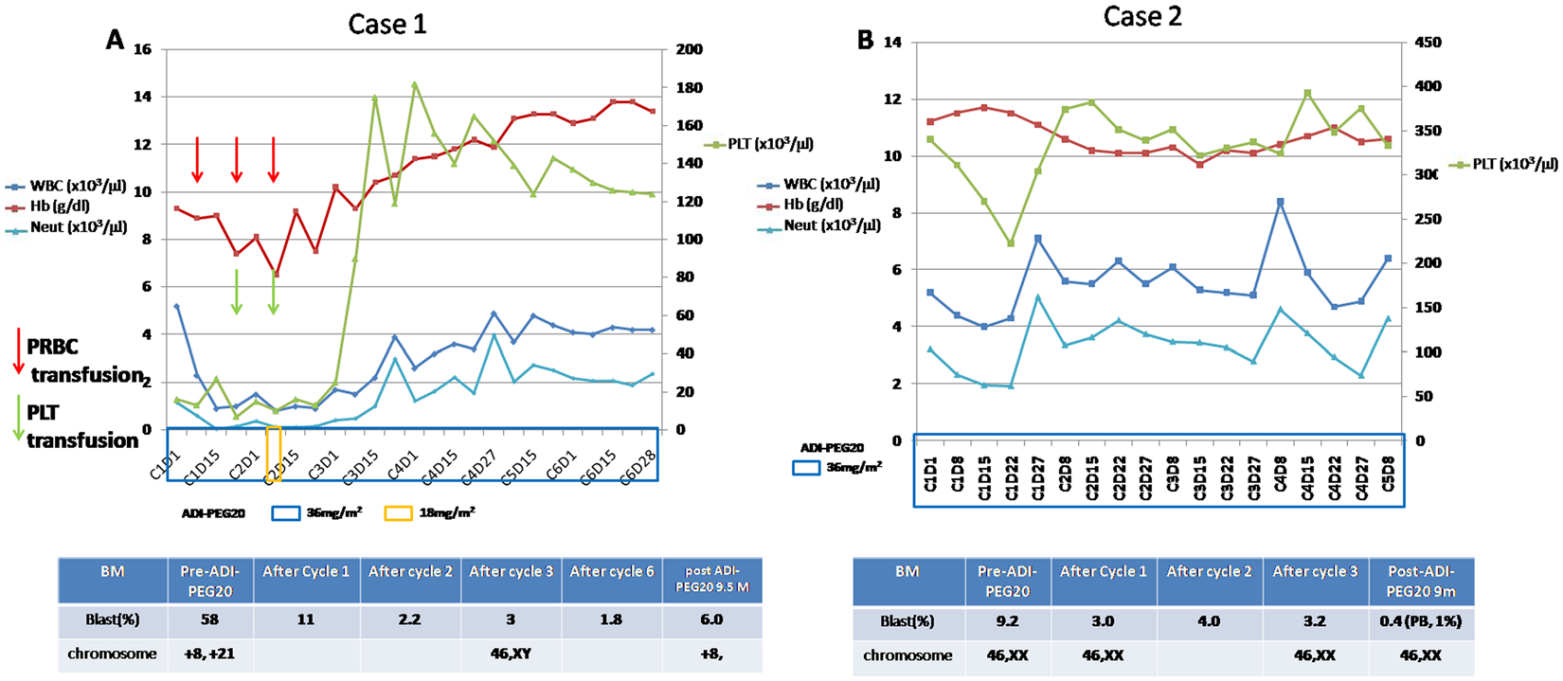
The complete blood count (CBC) and bone marrow (BM) profiles of Case 1 and Case 2 during ADI-PEG20 treatment. (**A**) The dynamic CBC and BM changes in Case 1 after ADI-PEG20 treatment and the timing of packed red blood cell and platelet transfusion. (**B**) The dynamic CBC and BM changes in Case 2 after ADI-PEG20 treatment.

Because there were 2 patients (Case 1 and Case 2) who had CR as a result of ADI-PEG20, we will describe their clinical courses. Case 1 was a 79-year-old man diagnosed with AML (M7) in July 2013. He received only supportive care prior to being accrued to this trial in February 2014. The patient developed itching maculo-papular skin eruptions over all four limbs at 2–3 days after the fifth weekly injection of ADI-PEG20; the eruptions were easily relieved by anti-histamines. The white blood cell (WBC) count decreased from 5200 to 900/μl, and blasts in peripheral blood (PB) decreased from 60% to 3% at two weeks after ADI-PEG20 injection.The dose of ADI-PEG20 was reduced to 18 mg/m^2^ upon the sixth injection due to leukopenia (WBC, 800/μl; absolute neutrophil count, 104/μl) but returned to 36 mg/m^2^ for all subsequent treatments. The blasts in BM decreased from a baseline of 58% before initiation of ADI-PEG20. The proportion decreased to 2.2% after two cycles of treatment (Fig. 3A). CR was achieved after 3 cycles of ADI-PEG20, and the chromosomes of BM were also converted from +8 and +21 to normal karyotypes. ADI-PEG20 was discontinued after 6 cycles. The circulating arginine level remained low until the 20th week post-ADI-PEG20 treatment, accompanied by the development of anti-ADI-PEG20 antibodies in the plasma. The patient relapsed in November 2014 with an increase in BM blasts to 6% noted in follow-up. The duration of response was 7.5 months. ADI-PEG20 was resumed without any response.

#### Case 2

was a 69-year-old woman diagnosed with AML (M2) in December 2012. She received low-dose Ara-C (LDAC), and CR was achieved in August 2013. LDAC was maintained, but AML relapse was noted in March 2014. The blasts in the BM in March 2014 were 10.8%. She received another 2 weeks of LDAC from March 27 to April 10, 2014, and the blasts in BM were 9.2% on May 5, 2014. The WBC, hemoglobin and platelet counts of the peripheral blood were within normal ranges. Due the lack of further response to LDAC at approximately one month later, she started receiving ADI-PEG20 in May 2014. CR was noted after one cycle of ADI-PEG20 with a decrease in BM blasts from 9.2% to 3% (Fig. 3B). The patient had a precipitous decrease in blood pressure in the fifth cycle of ADI-PEG20, and the treatment was stopped in October 2014. The hypotension was suspected to be anaphylactic, and it resolved in a few days without sequelae after administration of an inotropic agent. The patient remained in CR until March 2015. The duration of response was 8.8 months.

### Whole transcriptome RNA sequencing

**Figure 4:**
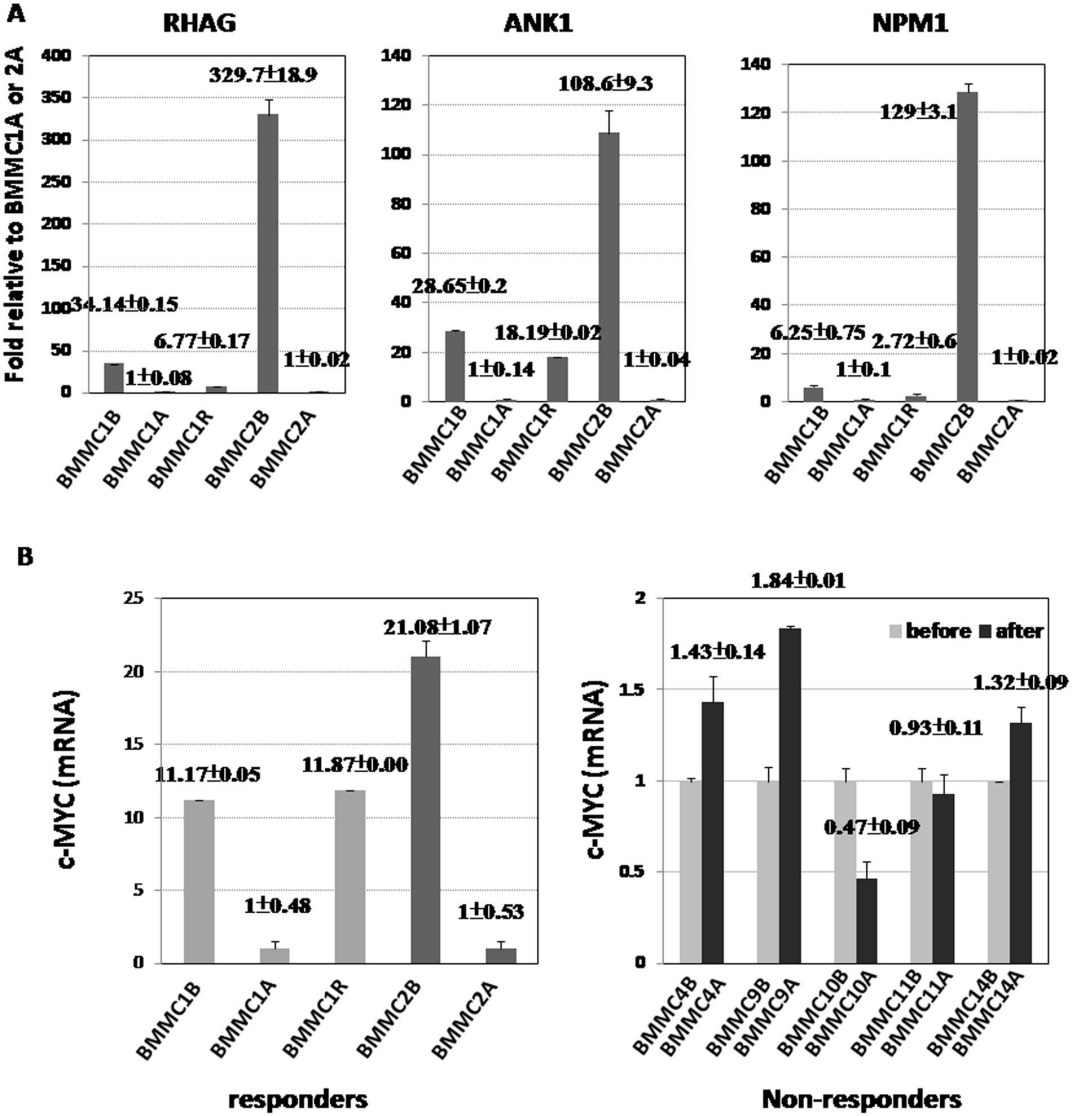
mRNA expression of associated genes in responders and non-responders before and after ADI-PEG20 treatment. (**A**) The relative mRNA expression levels of RHAG, ANK1, and NPM1 in the bone marrow mononuclear cells (BMMCs) of Case 1 and Case 2 at the indicated time points by QR-PCR. BMMC1B is the BMMCs of Case 1 before ADI-PEG20 treatment. BMMC1A is the BMMCs of Case 1 after ADI-PEG20 and in complete remission status. BMMC1R is the BMMCs of Case 1 collected at the time of relapse after ADI-PEG20 treatment. BMMC2B is the BMMCs of Case 2 before ADI-PEG20 treatment. BMMC2A is the BMMCs of Case 2 after ADI-PEG20 and in complete remission. The samples of BMMC1B and BMMC1R were compared with BMMC1A for analysis. The sample of BMMC2B was compared with BMMC1A for analysis. (**B**) The mRNA expression of c-MYC in AML cases responsive and non-responsive to ADI-PEG20. (left) Fold changes of mRNA expression of c-MYC in BMMC1B, and 1 R relative to BMMC1A and in BMMC 2B relative to BMMC2A. Case 1 and Case 2 were responders to ADI-PEG20. (right) Fold changes of mRNA expression of c-MYC in BMMC4A, 9 A, 10 A, 11 A, and 14 A relative to BMMC4B, 9B, 10B, 11B, and 14B, respectively. Cases 4, 9, 10, 11, and 14 were non-responders to ADI-PEG20. (Cases 4, 10, and 11were PD; Cases 9 and 14 were SD) “A” indicates after ADI-PEG20, “B” indicates before ADI-PEG20.

Whole transcriptome RNA sequencing (RNA-seq) for BM mononuclear cells (BMMCs) from the two CR patients (Cases 1 and 2) (M7 and M2) and another three non-responders (M0, M1, and CMMoL transformed AML) was performed, and gene set enrichment analysis^20^ was performed to identify the gene sets/pathways that were enriched in the differential expression profiles. Based on the quantitative analysis of transcripts in the RNA-seq data, significant global transcriptional changes were observed in the two CR patients between pre-ADI-PEG20 leukemic cells and post-ADI-PEG20 normal hematopoietic cells. Among the 21832 genes that were mapped from the samples, 10042 genes were up-regulated, and 8243 genes were down-regulated in pre-ADI-PEG20 treatment samples compared with post-ADI-PEG20 samples. For both cases, the gene sets related to markers of AML subtypes, M2, M4, M5 and M7 (ROSS_AML_OF_FAB_M7_TYPE and YAGI_AML_FAB_MARKERS), and the genes up-regulated in hematopoietic stem cells (JAATINEN_HEMATOPOIETIC_STEM_CELL_UP) were enriched in pre-ADI-PEG20 BMMCs versus post-ADI-PEG20 BMMCs, as shown in Supplementary Table 2. Some of the core genes enriched in pre-ADI-PEG20 BMMCs of these two responders are common genes on hematopoietic stem cells reported by other researchers^21^. These genes are underlined in Supplementary Table 2. Interestingly, down-regulation of c-MYC regulated genes was noted in both responders after ADI-PEG20. The genes in these gene sets, such as *TUBB4A, AKR1C1, AKR1C2, AKR1C3, RHAG, ANK1, GYPB, MYC, IMPDH2, APEX1*, and *NPM1*, were down-regulated in post-ADI-PEG20 BMMCs and up-regulated in the relapse of Case 1. Some of these genes were validated by qRT-PCR assay, shown in Fig. 4A. For Case 1, gene sets associated with RNA biogenesis, processing and metabolism and peptide chain elongation were significantly enriched in the BMMCs before ADI-PEG20. For Case 2, the enriched gene sets in the BMMCs before ADI-PEG20 treatment included PID_SYNDECAN_1_PATHWAY, NABA_COLLAGENS, and REACTOME_COLLAGEN_FORMATION, in addition to genes associated with AML markers. Taken together, these results indicated dramatic changes related to over-activation of corresponding pathways in the leukemia cells of the responders, likely during carcinogenesis.

The significantly enriched gene sets in the two responders versus the 3 non-responders before ADI-PEG20 treatment included those associated with markers of AML subtypes and the platelet aggregation plug pathway (Supplementary Table 3). Other significantly up-regulated gene sets identified in only the responders included those related to STAT5^22^, E2F3^23^, Wnt and TNFα signaling, represented by up-regulation of the genes *RYR3, RHAG, ANK1, PTGER3, GP1BB*, *GAD1*, etc. By contrast, the significantly up-regulated gene sets identified in the non-responders included SCHURINGA_STAT5A_TARGETS_DN, VALK_AML_CLUSTER_10, KAMIKUBO_MYELOID_CEBPA_NETWORK, and those related to the CEBPA network, NOTCH1, AKT1, FLT3-ITD, oxidative stress response and leukemic stem cell signaling, as shown in Supplementary Table 3.

According to the RNA-seq results, a total of 1215 and 950 variants were identified in the BMMCs of Cases 1 and 2, respectively, before ADI-PEG20 treatment. Fifty-eight and 7 non-synonymous mutations were noted in Cases 1 and 2, respectively. Fourteen of the 58 non-synonymous mutations were predicted to be damaged or deleterious by PPH2 (PolyPhen)^24^ or SIFT^25^ analysis in Case 1, including *LUZP1*, *MRPL37*, *TSPYL1*, *BRAT1*, *NAB2*, *RNF31*, *IPO4*, *CEP152*, *PYGB*, *TUBGCP6*, *CAPN2*, *XRRA1*, *SPSB2*, and *GP1BA* (Supplementary Fig. 1). For Case 2, no mutations were predicted to be deleterious by PPH2 or SIFT in BMMC2B but in BMMC2A. The mutations with changes in protein coding in Case 2, BMMC2B, are listed in Supplementary Fig. 1. Some of the mutations in Cases 1 and 2 were validated by Sanger sequencing. Compared with the 3 non-responders, the mutations in *MRPL37*, *CEP152*, *PYGB*, *TUBGCP6*, *CAPN2*, *XRRA1*, and *SPSB2* were unique to Case 1. Three mutations (*SNX18, TSPYL1, and BRAT1*) were detected at the time of relapse for Case 1.

## Discussion

In this study, we showed that single-agent ADI-PEG20 therapy resulted in a CR rate of 4.7% (95% CI, 0~11.2%) and a stable disease rate of 16.3% (95% CI, 4.8~27.8%) among 43 relapsed/refractory or poor-risk AML patients with response durations of 7.5 and 8.8 months for two patients achieving CR. The duration of arginine depletion, measured 8 days after the prior ADI-PEG20 dose, was correlated with disease control with a median of 8 weeks of suppression to ≤10 μM. This result was consistent with the arginine starvation mechanism of action of ADI-PEG 20; the tumors depleted of arginine for the longest time had the best responses. The most common toxicity was grade 1/2 skin rash, which occurred in only 4 patients (9.3%). The most severe adverse event that occurred was grade 4 tumor lysis syndrome in one case and grade 4 anaphylactic shock in another. The patient who developed tumor lysis syndrome after ADI-PEG20 died of acute renal failure and respiratory failure due to pneumonia with a baseline neutropenia status. The patient who had anaphylactic shock after ADI-PEG20 recovered after inotropic agent treatment without any sequelae. Compared with chemotherapy, the hematologic and non-hematologic toxicity of ADI-PEG20 was minimal^3, 4^. The safety profile of ADI-PEG20 supported that it is suitable for treating elderly AML patients. However, the occurrence of suspected anaphylactic shock must be acknowledged. ADI is a non-human protein, and such hypersensitivity adverse events are well known with biologic agents^26^.

In this study, the leukemia cells of almost all of the patients were ASS-deficient, as assessed either by western blot or by immunohistochemistry (data not shown). Based on this knowledge, we expected higher effectiveness of ADI-PEG20 monotherapy in this patient population.

ADI-PEG20 has been tested for its toxicity and response in phase I/II studies for melanoma, hepatocellular carcinoma and mesothelioma^15–17, 27^. According to the published results, the majority of patients achieved SD, with a disease control rate that ranged from 31% to 63% in the three phase II studies, including two HCC studies and one melanoma study. In the HCC study reported by Glazer *et al*., one patient achieved CR and one patient achieved PR among the 76 evaluable patients^15^. Development of anti-ADI-PEG20 antibodies with concurrent elevation of circulating arginine levels in patients is a potential tumor escape mechanism for ADI-PEG20. In our study, the circulating arginine and citrulline levels and anti-ADI-PEG20 antibodies in AML patients were similar to those in HCC patients treated with ADI-PEG20 monotherapy. However, the responses of individual patients to ADI-PEG20 treatment in this trial were not predictable based on baseline ASS levels, as the hypothesis suggested. Initial decreases in circulating arginine levels were observed in all the patients who received ADI-PEG20. The two CR patients had elevated arginine levels detected at the 20th and 8th weeks after ADI-PEG20 for Case 1 and Case 2, respectively, but both remained in CR for more than 7 months. Nine of 12 PD patients among 21 evaluable patients had persistent low arginine levels at the same time of evaluation as PD. The results suggested that other factors in addition to arginine deprivation are needed to achieve a response in AML patients treated with ADI-PEG20 monotherapy.

Similar results have been noted with the amino acid deprivation agent asparaginase in acute lymphocytic leukemia, with improved results observed by combining it with a cytotoxic agent^28^. Similarly, in preclinical studies, ADI-PEG20 has been combined with cytarabine^19^. ADI-PEG20 alone induced responses in 19 of 38 AML cases *in vitro* and 3 of 6 AML cases *in vivo*, leading to caspase activation in sensitive AMLs. ADI-PEG20-resistant AMLs showed higher relative expression of ASS1 than sensitive AMLs. This finding suggested that the resistant AMLs survived by producing arginine through this metabolic pathway, and ASS1 expression could be used as a biomarker for response. Sensitive AMLs showed more robust uptake of arginine from the extracellular environment, consistent with their auxotrophy for arginine. The combination of ADI-PEG20 and cytarabine chemotherapy was more effective than either treatment alone, resulting in responses in 6 of 6 AML cases tested *in vivo*. As a result of this arginine addiction, further clinical trials of ADI-PEG20 in AML have been recommended^29^. Such a combination therapy has already been recommended for ADI-PEG20 in solid tumors^9, 30^.

Using RNA sequencing, we found that genes associated with AML subtypes, such as *RYR3, RHAG, PCDH9, ANK1, ADAMTS3*, and *DLC1*, were significantly up-regulated in the leukemia cells of two responders, but not in the 3 non-responders. The presence of these markers could be helpful in predicting subgroups of AML patients who are potentially more responsive to ADI-PEG20. Down-regulation of genes associated with STAT5A and c-MYC was noted in the BMMCs of both responders after ADI-PEG20 treatment. We compared the c-MYC expression in BMMCs before and after ADI-PEG20 in the two responders (Cases 1 and 2) and in 5 other non-responders (Cases 4, 9, 10, 11, and 14), including 2 SD (Case 9 and Case 14) and 3 PD patients, by qRT-PCR. The c-MYC expression level was down-regulated in Cases 1 and 2 after ADI-PEG20 treatment, as shown in Fig. 4B, whereas the c-MYC expression was not decreased in Cases 4, 9, 11 or 14 after ADI-PEG20 treatment. Additionally, elevation of c-MYC was noted in the relapsed BMMCs in Case 1 (Fig. 4B). Further investigation to determine whether ADI-PEG20 inhibits AML proliferation by targeting c-MYC and whether the expression level of c-MYC could serve as a predictor of the effectiveness of ADI-PEG20 treatment is worthwhile. In the two responders, we did not find identical mutations in the BMMCs before ADI-PEG20 treatment. Although we did identify 7 unique mutations (*MRLP37*, *CEP152*, *PYGB*, *TUBGCP6*, *CAPN2*, *XRRA1*, and *SPSB2*) among the 14 potentially deleterious genetic mutations in Case 1 before ADI-PEG20 treatment (Supplementary Fig. 1), these mutations were not frequently reported in AML patients, and they were not found in the non-responders or in the normal hematopoietic cells from Case 1. The effects of these gene mutations on the response to arginine deprivation require further investigation.

In conclusion, weekly ADI-PEG20 monotherapy resulted in a low response rate among patients with refractory/relapsed or poor-risk AML with minimal toxicities. This finding was associated with the suppression of peripheral blood arginine. Interestingly, two patients with AML had relatively durable CR after ADI-PEG20 treatment. Further investigations are warranted to explore the biological mechanism of ADI-PEG20 in AML, the biological markers of response and potential combinations, including with cytarabine, for the treatment of AML.

## Patients and Methods

### Eligibility criteria

This study was sponsored by Polaris Group and registered with ClinicalTrials.gov as NCT01910012 on 10/07/2013. Patients ≥ 18 years old with relapsed/refractory or poor-risk AML and not candidates for stem cell transplantation were eligible. Poor-risk AML included treatment-related AML, antecedent hematologic disease (e.g., myelodysplastic syndrome, myelofibrosis, and polycythemia vera), unfavorable cytogenetics regardless of age and *de novo* AML at ≥60 years of age. Patients who had uncontrolled concomitant illness including, but not limited to, ongoing or active infection, symptomatic congestive heart failure (New York Heart Association Class III or IV), cardiac arrhythmia, psychiatric illness, social situations that would limit compliance with study requirements, disseminated intravascular coagulation or a history of another active primary cancer, except curatively resected skin cancer or treated cervical carcinoma *in situ*, were excluded. The study was approved by the institutional review board of each participating institution, and all of the patients provided signed informed consent. In addition, this trial was approved by the Institutional Review Boards of National Cheng Kung University Hospital, Tainan, Taiwan; Kaohsiung Medical University Hospital, Kaohsiung, Taiwan; University of Texas M.D. Anderson Cancer Center, Houston, TX, USA; Kaohsiung Chang Gung Memorial Hospital, Kaohsiung, Taiwan; Linkou Chang Gung Memorial Hospital, Linkou, Taiwan; China Medical University Hospital, Taichung, Taiwan and performed in accordance with their guidelines and regulations of each participating hospital.

### Treatment and evaluation

The patients received weekly intramuscular injections of ADI-PEG20 36 mg/m^2^ (4 weeks as one cycle). Patients with platelet counts <20 × 10^9^/l received platelet transfusions prior to administration of ADI-PEG20. Prophylactic use of allopurinol was permitted for the prevention of hyperuricemia. The tumor response was assessed according to the revised recommendations of the International Working Groups^31, 32^ .The patients who did not fulfill the response criteria for partial response or progressive disease were classified as having stable disease (SD). Bone marrow aspiration was performed at the time of enrollment and after the second and third cycles of treatment to evaluate the response. Additional BM aspirations were to be performed after the first cycle of treatment if the complete blood count (CBC) showed evidence of a significant response after the first cycle or anytime at the discretion of the investigator. Treatment was continued until the occurrence of disease progression, development of unacceptable toxicity, death, or withdrawal of consent for any reason. If the patients achieved CR or CR with incomplete blood count recovery (CRi), the treatment was completed after another 4 cycles of ADI-PEG20. The CBC, differential count, biochemistry, and toxicities were evaluated at each visit or at the discretion of the investigator. The severity of all adverse events was assessed according to the NCI CTCAE Scale, version 4.0. Pharmacodynamic and immunogenicity parameters, including circulating levels of arginine and citrulline and antibodies to ADI-PEG20 were measured on days 1, 8, 15, 22, and 27 of cycle 1 and day 1 of cycles 2–6. Thus, these parameters were assessed 8 days after the prior ADI-PEG20 dose. Circulating arginine and citrulline levels were measured by mass spectrometry assays, and plasma anti-ADI-PEG20 antibodies were assessed by ELISA-based immunogenicity assay. Arginine depletion was defined as ≤10 μM. These tests were performed by Polaris Pharmaceuticals, Inc. (San Diego, CA, USA). Determination of BM ASS was recommended but not required prior to enrollment and upon BM follow-up. ASS was determined as described previously, using a Polaris antibody.

### Statistical consideration

The primary endpoint was the response rate. The second endpoints were safety, tolerability, time of treatment, pharmacodynamics and immunogenicity of ADI-PEG20 and the overall survival (OS) of the patients. Data, including the safety, pharmacodynamics and immunogenicity of ADI-PEG20, were analyzed according to the intention-to-treat (ITT) principle. For the efficacy evaluation, the per-protocol evaluable population was analyzed. The evaluable patients were those who were enrolled in this study and who received at least two doses of the study drug during the first two weeks on a consecutive basis and also who a completed baseline tumor evaluation and at least one post-baseline tumor evaluation. The lack of activity hypothesis (<5% response rate) was to be accepted if 2 or fewer responses were seen with an 82% power for an alternative response rate of 20% at an 8%, one-sided significance level. Therefore, a total of up to 21 evaluable patients were to be enrolled in the study. The Clopper-Pearson exact method was used to calculate the 95% confidence interval (C.I.) for the response rate. The survival distributions were estimated using the Kaplan-Meier method.

### RNA-seq analysis and GSEA analysis

RNA-seq analysis of some of the enrolled patients was approved by the Institutional Review Board of National Cheng-Kung University Hospital (Tainan, Taiwan), and the patients provided signed informed consent forms. RNA-seq was performed using an Ion Proton System (Thermo Fisher Scientific, Waltham, MA, USA) at the Center for Genomic Medicine, National Cheng Kung University, according to the manufacturer’s instructions. Briefly, approximately 1 μg of total RNA for each sample was subjected to whole transcriptome library preparation using an Ion Total RNA-Seq Kit v2 (Thermo Fisher Scientific, Waltham, MA, USA). The samples were processed using a One Touch 2 instrument, followed by enrichment with a One Touch ES station (Life Technologies), and they were then sequenced with the Ion Proton Sequencer using an Ion PI chip v2 (Thermo Fisher Scientific, Waltham, MA, USA), according to the manufacturer’s instructions.

The FASTQ files obtained from the Ion Proton sequencing system were then mapped to the reference genome (Human, hg19) using TopHat2^33^ and Bowtie2^34^. The merged mapping results in BAM files were then subjected to gene expression analysis, using Partek software for alignment, gene mapping, and normalization. The quantile normalized RPKM (read per kilobase per million) value of all of the mapped genes was subsequently subjected to GSEA analysis. Before GSEA analysis, the genes with zero counts in both samples were first removed. For each of the remaining genes, the ratio of normalized RPKM after treatment to that before treatment was then obtained, and the logarithm (with base 2) of that ratio was input into GSEA analysis using the algorithm of log2_ ratio_ of_ class for the canonical pathway (cp) and chemical and genetic perturbation (cgp) gene set analysis.

## Electronic supplementary material

Supplementary Tables and Figure

## References

[1] Döhner H (2015). Acute myeloid leukemia. N Engl J Med.

[2] Health Promotion Administration, Ministry of Health and Welfare, Taiwan Health Promotion Administration Annual Report. Available from: http://www.hpa.gov.tw/EngPages/List.aspx?nodeid=1072. Date of access: 10/05/2017 (2016).

[3] Hengeveld M (2012). Intensive consolidation therapy compared with standard consolidation and maintenance therapy for adults with acute myeloid leukaemia aged between 46 and 60 years: final results of the randomized phase III study (AML 8B) of the European Organization for Research and Treatment of Cancer (EORTC) and the Gruppo Italiano Malattie Ematologiche Malignedell ‘Adulto (GIMEMA) Leukemia Cooperative Groups. Ann Hematol.

[4] Jehn U (2006). Non-infusional vs intravenous consolidation chemotherapy in elderly patients with acute myeloid leukemia: final results of the EORTC-GIMEMA AML-13 randomized phase III trial. Leukemia.

[5] Patel JP (2012). Prognostic relevance of integrated genetic profiling in acute myeloid leukemia. N Engl J Med.

[6] Knapper S (2006). A phase 2 trial of the FLT3 inhibitor lestaurtinib (CEP701) as first-line treatment for older patients with acute myeloid leukemia not considered fit for intensive chemotherapy. Blood.

[7] Jain N (2014). Phase II study of the oral MEK inhibitor Selumetinib in advanced acute myeloid leukemia: a University of Chicago phase II consortium trial. Clin Cancer Res.

[8] Garcia-Manero G (2015). Phase I dose escalation trial of ilorasertib, a dual Aurora/VEGF receptor kinase inhibitor, in patients with hematologic malignancies. Invest New Drugs.

[9] Phillips MM (2013). Targeting arginine-dependent cancers with arginine-degrading enzymes: opportunities and challenges. Cancer Res Treat.

[10] Schimke RT (1966). The generation of energy by the arginine dihydrolase pathway in Mycoplasma hominis 07. J Biol Chem.

[11] Tytell AA (1960). Growth response of stable and primary cell cultures to L-ornithine, L-citrulline, and L-arginine. Exp Cell Res.

[12] Delage B (2012). Promoter methylation of argininosuccinate synthetase-1 sensitises lymphomas to arginine deiminase treatment, autophagy and caspase-dependent apoptosis. Cell Death Dis.

[13] Ensor CM (2002). Pegylated arginine deiminase (ADI-SS PEG20,000 mw) inhibits human melanomas and hepatocellular carcinomas *in vitro* and *in vivo*. Cancer Res.

[14] Kim RH (2009). Arginine deiminase as a novel therapy for prostate cancer induces autophagy and caspase-independent apoptosis. Cancer Res.

[15] Glazer ES (2010). Phase II study of pegylated arginine deiminase for nonresectable and metastatic hepatocellular carcinoma. J Clin Oncol.

[16] Feun LG (2012). Negative argininosuccinate synthetase expression in melanoma tumours may predict clinical benefit from arginine-depleting therapy with pegylated arginine deiminase. Br J Cancer.

[17] Yang TS (2010). A randomised phase II study of pegylated arginine deiminase (ADI-PEG 20) in Asian advanced hepatocellular carcinoma patients. Br J Cancer.

[18] Szlosarek P. *et al*. Pegylated arginine deiminase (ADI-PEG20) as a potential novel therapy for argininosuccinatesynthetase-deficient acute myeloid leukemia. In: Proceedings of the 102nd Annual Meeting of the American Association for Cancer Research-Innovation and Collaboration: The Path to Progress; 2011 Apr 2-6; Orlando, FL Philadelphia (PA): AACR; *Cancer Res* 2011;**71(**8 Suppl**)**, Abstract nr 4067. doi:10.1158/1538-7445.AM2011-4067.

[19] Miraki-Moud F (2015). Arginine deprivation using pegylated arginine deiminase has activity against primary acute myeloid leukemia cells *in vivo*. Blood.

[20] Subramanian A (2005). Gene set enrichment analysis: a knowledge-based approach for interpreting genome-wide expression profiles. Proc Natl Acad Sci USA.

[21] Jaatinen T (2006). Global expression profile of human cord blood-derived CD133+ cells. Stem Cell.

[22] Schuringa JJ (2004). Constitutive activation of STAT5A promotes human hematopoietic stem cell self-renewal and erythroid differentiation. J Exp Med.

[23] Olsson AY (2007). Role of E2F3 expression in modulating cellular proliferation rate in human bladder and prostate cancer cells. Oncogene.

[24] Adzhubei IA (2010). A method and server for predicting damaging missense mutations. Nat Methods.

[25] Kumar P (2009). Predicting the effects of coding non-synonymous variants on protein function using the SIFT algorithm. Nat Protoc.

[26] Corominas M (2014). Hypersensitivity reactions to biological drugs. J Investig Allergol Clin Immunol.

[27] Szlosarek PW (2013). Metabolic response to pegylated arginine deiminase in mesothelioma with promoter methylation of argininosuccinatesynthetase. J Clin Oncol.

[28] Vadlamudi S (1971). Studies on neutralization of L-asparaginase activity *in vitro* and *in vivo*. Cancer.

[29] Plunkett W (2015). Arginine addiction in AML. Blood.

[30] Patil MD (2016). Arginine dependence of tumor cells: targeting a chink in cancer’s armor. Oncogene.

[31] Cheson BD (2003). International Working Group for Diagnosis, Standardization of Response Criteria, Treatment Outcomes, and Reporting Standards for Therapeutic Trials in Acute Myeloid Leukemia.Revised recommendations of the International Working Group for Diagnosis, Standardizationof Response Criteria, Treatment Outcomes, and Reporting Standards for Therapeutic Trials in Acute Myeloid Leukemia. J Clin Oncol.

[32] Sekeres MA (2011). A phase 2 study of lenalidomidemonotherapy in patients with deletion 5q acute myeloid leukemia: Southwest Oncology Group Study S0605. Blood.

[33] Kim D (2013). TopHat2:accurate alignment of transcriptomes in the presence of insertions, deletions and gene fusions. Genome Biol.

[34] Langmead B (2012). Fast gapped-read alignment with Bowtie 2. Nat Methods.

