# Supplementary material for: A Phase II Study of Arginine Deiminase (ADI-PEG20) in Relapsed/Refractory or Poor-Risk Acute Myeloid Leukemia Patients
Source: Sci Rep. 2017 Sep 12;7:11253. doi: 10.1038/s41598-017-10542-4 (PMC5595917; doi:10.1038/s41598-017-10542-4)
Supplement: Supplementary file 1 — Supplementary Tables and Figure [file 41598_2017_10542_MOESM1_ESM.pdf]

A Phase II Study of Arginine Deiminase (ADI-PEG20) in Relapsed/Refractory or Poor-Risk Acute Myeloid Leukemia Patients

Hui-Jen Tsai[1,2,3], Shih Sheng Jiang[1], Wen-Chun Hung[1], Gautam Borthakur[4], Sheng-Fung Lin[3], Naveen Pemmaraju[4], Elias Jabbour[4], John S. Bomalaski[5], Ya-Ping Chen[2], Hui-Hua Hsiao[3], Ming-Chung Wang[6], Ching-Yuan Kuo[6], Hung Chang[7], Su-Peng Yeh[8], Jorge Cortes[4], Li-Tzong Chen[1,2,3,9], Tsai-Yun Chen[2]

Supplementary Table 1. FAB subtype, baseline marrow blast percentage and cytogenetic change of the 21 evaluable patients

Cases	CR+SD (N=9)			Cases	PD (N=12)		
	FAB subtype	baseline BM blast (%)	cytogenetic abnormality		FAB subtype	baseline BM blast (%)	cytogenetic abnormality
1	M7	58.2	+8, +21	3	M2	71	normal
2	M2	9.2	normal	4	M0	76.2	del(18), -18
6	M0	70-80	43-47. XX, add(18)	5	M2	58	t(8;21)
9	M4eo	44.6	inv(16)	7	M4	>90	del(15)
12	M0	64.2	+11, +14, +2, +21, add(7)(p22), -12	8	M1	81.5	der(7;12)
14	M0	50.8	del(9)	10	M2	63.6	del(1), -5, -6, -8, -9, add(15, add(16), -18, +21, +6mar(16))
16	N/A*	59	+1. add(1), del(1), add(2), del(3)	11	M1	72.8	del(1), ad(2), del(5), add(16), -17, -18
17	N/A*	34	del(6q)	13	M1	89.6	del(2), del(5), +6, add(6), +8, -18, idem, +20, -7, add(14)
18	N/A*	30	normal	15	N/A*	46	t(1;3), t(1;6), der(3)t(3;10)
				19	N/A*	15	del(13), del(20), +Y
				20	N/A*	14	normal
				21	N/A*	26	inv(16), del(20)

median**		50.8				67.3	

*, N/A: not available

**, *P*= 0.25 by Wilcoxon Rank Sum Test

Supplementary Table 2

enriched gene sets in BMMC of Case 1 and Case 2 before ADI-PEG20 versus after ADI-PEG20

Gene set	nominal p-value	FDR q-value	NES*	core genes
ROSS_AML_OF_FAB_M7_TYPE	<0.001	<0.001	-3.08	DLC1, RHAG, RYR3, GATA2, PLOD2, ITGA2B, KEL, LAPTM4B, TFR2, PROS1, PNMT, MINPP1, DNM3, ANK1, SERPINI1, TAL1, APOE, DNAJC6, PCDH9, PDLIM5, MYH10, ABCC4, NET1, NEO1, FADS2, UROD, GP1BA, BMP2K, KCNH2, NGFRAP1, CMAS, GATA1, TIMP3, KLF1, HLTF, FHL2, ICAM4, DNAJC9, TPM1, SDPR, MYL4, RDX, CTNNBL1, PRUNE, PDCD10, PCCB, APOC1, ZMYND8, ALDH1A1
*JAATINEN_HEMATOPOIETIC_STEM_CELL_UP	<0.001	<0.001	-2.58	MEG3, MPL, SCN3A, GATA2, MYCT1, VWDE, OBSL1, DEPTOR, LAPTM4B, KIAA1211, CYTL1, PDZD2, RBPMS, FAM92A1, CPA3, TANC1, FAM69B, ZBTB8A, MAST4,

				ZNF711, KIT, SMARCA1, C11orf95, ZNF521, GNAI1, CBX2, NDN, ANGPT1, SHANK3, SCHIP1, CRISPLD1, TFPI, PTPLA, MAP7, PREX2, CALN1, SEPP1, DPY19L2, HMGA2, TXNRD3, FGD5, FAM171B, CNKSR3, COL24A1, GCSH, LOC100506844, ME3, LIMCH1, SERPING1, FRMD6, KHDRBS3, NKAIN2, ARHGAP22, AKR1C3, SOCS2, ZC3HAV1L, SMAD1, BCAT1, GUCY1A3, CDK6, TRO, CRHBP, GPR126, DDAH1, GATM, RHOBTB1, PDGFC, CD109, NPR3, MEIS1, JUP, ISYNA1, TMEM200A, MEST, ZBED3, FAIM, CD34, PGBD1, MYB, MAP9, PON2, DNMT3B, MBLAC2, TRIP6, PXDN, TMEM5, KDELC1, COL5A1, SLC39A8, PLA2G12A, TRIM24, CHST13, TMEM44, ZNF618, FHL1, HDGFRP3, BEND4, ZNRF1, CCNB1IP1, MYO5C, DPPA4,

				ALG10B, STMN1, TAF1D, PPM1H, GPR125, KDM5B, ATP2C1, ANKRD28, DPY19L4, SRD5A3, DOCK7, PLEKHA5, FAM115A, RDX, ATP6V0A2, TUSC1, CDK2AP1, SCN9A, BIVM, EBPL, PAIP1, GOLIM4, C11orf54, FAM175A, ALDH1A1, PROM1, DTL, TMEM38B, TSPYL5, PRMT5, F2RL1, ZNF165, SLC39A10, BSPRY, WDR17, PAICS, CDK4, TRIM73, FABP5, HSPD1, WBP5, CPT1A, NT5DC2, ANKRD6, PSMB5, SSBP2, TNFRSF21, WASF1, CMAHP, ZNF512B, HOXA9, IGLL1
YAGI_AML_FAB_MARKERS	<0.001	0.003	-2.07	MYO16, GABRE, DLC1, FGFR3, GAD1, RHAG, PYR3, GATA2, ADAMTS3, TRPC6, PLOD2, ITGA2B, KEL, PBX1, LAPTM4B, PROS1, CFH, LAMA5, CXADR, MAST4, SERPINI1, CDC42BPA, TRPC1, SERPINE2, HERC2P2, PIR, CD200, MYH10, PKIA, NET1,

				ALDH5A1, TRIB2, GP1BA, ULK2, STAC, PKIG, SERPINH1, MLLT11, UBXN8, BAMBI, CAV1, ELOVL6, TIMP3, CLCN4, LDB1, ZBTB16, CD180, BAG2, SNTA1, HDGFRP3, TPM1, DLEU1, MYL4, RFC3, TNFRSF4, FADS1, TFDP2, ACO1, PAQR3, PDCD10, TGM2, FAM20B, PCCB, DDHD2, NAE1, GP9, ZMYND8, ALDH1A1, TBPL1
SCHURINGA_STAT5A_TARGETS_UP	<0.001	0.005	-2.02	GYPB, RHAG, HBE1, ANK1, CD36, LEPR, XK, KLHL29, PIM1, CISH
REACTOME_PLATELET_AGGREGATION_PLUG_FORMATION	0.006	0.133	-1.69	MPL, GP1BB, ADRA2A, ITGA2B, RAPGEF3, GP1BA, GP5, PTK2, VWF, RAPGEF4, FN1, GP9
REACTOME_FORMAT	0.038	0.197	-1.56	TUBB4A, CCT2, TUBA1C, TUBB2B, TUBB6, TUBA1A, CCT3, TUBA1B,

ION_OF_TUBULIN_FOLDING_INTERMEDIATES_BY_CCT_TRIC				TUBB2A, CCT4, TCP1, TUBB3, TUBB4B, CCT6A, CCT7, CCT5
HALLMARK_MYC_TARGETS_V2	0.024	0.127	-1.48	MYC, NDUFAF4, RCL1, SORD, BYSL, NPM1, GNL3, PA2G4, CDK4, HSPD1, RABEPK, DCTPP1, PLK4, MCM4, NOLC1, PLK1, PRMT3, MRTO4, IPO4, UNG, NIP7, SLC29A2, TBRG4, PUS1, PPAN, SRM, TFB2M, DDX18, TCOF1, WDR74, TMEM97, RRP9, MYBBP1A, UTP20, AIMP2, CBX3, NOP16, PPRC1, WDR43, MPHOSPH10, NOP2
HALLMARK_MYC_TARGETS_V1	0.068	0.322	-1.23	MYC, IMPDH2, CCT2, APEX1, HSP90AB1, ODC1, NPM1, CCT3, CDC20, HNRNPA1, TFDP1, EIF4E, GNL3, POLD2, GLO1, PA2G4, CDK4, MAD2L1, HSPD1, RAN,

				PRM1, SET, RPS3, ACP1, ILF2, MCM4, NOLC1, IARS, CCT4, EIF2S1, TOMM70A, HNRNPC, CDK2, SMARCC1, YWHAQ, TCP1, GSPT1, YWHAE, ABCE1, RPS6, G3BP1, RPL14, ORC2, NCBP2, POLE3, DEK, GNB2L1, HDGF, SRSF3, HDAC2, ERH, CCT7, SF3B3, EIF4G2, RSL1D1, EIF3J, PPIA, CCT5, MCM7, EIF4A1, RPL22, VDAC3, C1QBP, TRIM28, SRSF7, SYNCRIP, RPS2, PWP1, RPLP0, SNRPG, CCNA2, PSMD14, XPO1, SRM, MRPL9, RFC4, HNRNPR, DHX15, TUFM, EPRS, DDX18, SERBP1, BUB3, CANX, KARS, MCM2, SSB, CAD, PTGES3, RRP9, PSMA1, PABPC4, RPS10, PSMA4, SLC25A3, AIMP2, EXOSC7, SSBP1, PHB2, HNRNPA2B1, DDX21, H2AFZ, TRA2B, PSMB2, CLNS1A, CBX3, EIF3B, PRDX3, EEF1B2, HNRNPU, NME1, NOP16, VDAC1,

				USP1, EIF4H, PPM1G, UBE2E1

*____, the common genes on hematopoietic stem cells

enriched gene sets in BMMC of Case 1 before ADI-PEG20 versus after ADI-PEG20

Gene set	nominal p-value	FDR q-value	NES*	Core genes
ROSS_AML_OF_FAB_M7_TYPE	<0.001	<0.001	-3.21	DLC1, PLOD2, RYR3, LAPTM4B, GATA2, PNMT, ITGA2B, TFR2, RHAG, DNM3, KEL, STXBP6, PCDH9, PROS1, PDLIM5, SERPINI1, MINPP1, APOE, ANK1, TIMP3, NGFRAP1, NET1, ABCC4, FADS2, TAL1, SLC39A4, DNAJC6, MYH10, GATA1, BMP2K, NEO1, ICAM4, KLF1, KCNH2, CTNNBL1, GP1BA, ZMYND8, MYL4, APOC1, SDPR, TPM1, PCCB, HLTF, PTGS1, ARMC8, CMAS, PDCD10, C14orf2, DRAP1, MRPS12, DNAJC9, RDX, UROD, ALDH1A1, PSMD6, PRUNE, SOD1, SQLE
KEGG_RIB	<0.001	<0.001	-2.56	RPL36A, RPL31, RPS3, RPS8,

OSOME				RPS24, RPL9, RPS17, RPS3A, RPL12, RPS4X, RPL22L1, RPL7, RPL36, RPS20, RPS6, RPS5, RPL3, RPL10A, RPS18, RPLP0, RPS21, RPL15, RPL29, RPS23, RPL24, RPL37A, MRPL13, RPL14, RPL7A, RPL5, RPS11, RPS26, RPL8, RPL32, RPS2, RPS12, RPL4, RPL41, RPL22, RPL27A, RPS27A, RPL18A, RPS29, RPL35, RPL13A, RPL37, RPL10, RPS9, RPL18, RPSA, RPS4Y1, RPL30, RPS10, RPL13, RPS7, RPS15, RPL23, RPS25, RPS27, RPL19, RPL26, RPL23A, RPL34, RPL11, RPL35A, RPL28, RPL17, RPS19
REACTOME_PEPTIDE_CHAIN_ELONGATION	<0.001	<0.001	-2.56	RPL36A, RPL31, RPS3, RPS8, RPS24, RPL9, RPS17, RPS3A, RPL12, EEF2, RPS4X, RPL7, RPL36, RPS20, RPS6, RPS5, RPL3, RPL10A, RPS18, RPLP0, RPS21, RPL15, RPL29, RPS23, RPL24, RPL37A, RPL14, RPL7A, RPL5, RPS11, RPS26, RPL8, RPL32, RPS2, RPS12,

				RPL4, RPL41, RPL22, RPL27A, RPS27A, RPL18A, RPS29, RPL35, RPL13A, RPL37, RPL10, RPS14, RPS9, RPL18, RPSA, RPS4Y1, RPL30, RPS10, RPL13, RPS7, RPS15, RPL23, RPS25, RPS27, RPL19, RPL26, EEF1A1, RPL23A, RPSAP9, RPL34, RPL11, RPL35A, RPL28, RPL17, RPL19
REACTOME_3_UTR_MEDIATED_TRANSLATIONAL_REGULATION	<0.001	<0.001	-2.53	RPL36A, RPL31, RPS3, RPS8, RPS24, RPL9, RPS17, RPS3A, RPL12, EIF3E, RPS4X, RPL7, RPL36, RPS20, RPS6, RPS5, RPL3, RPL10A, RPS18, RPLP0, EIF3D, RPS21, RPL15, EIF3C, RPL29, RPS23, RPL24, RPL37A, RPL14, RPL7A, RPL5, RPS11, RPS26, RPL8, RPL32, RPS2, RPS12, RPL4, EIF3H, RPL41, EIF3B, RPL22, RPL27A, RPS27A, RPL18A, RPS29, EIF4G1, RPL35, RPL13A, RPL37, RPL10, RPS14, RPS9, RPL18, EIF1AX, RPSA, EIF3J, RPS4Y1, RPL30,

				RPS10, RPL13, EIF3F, RPS7, RPS15, RPL23, RPS25, RPS27, RPL19, EIF4A1, RPL26, RPL23A, RPSAP9, RPL34, EIF4E, RPL11, RPL35A, RPL28, RPL17, RPS19, RPL6, EIF4B
REACTOME_FORMATION_OF_THE_TERNARY_COMPLEX_AND_SUBSEQUENTLY_THE_43S_COMPLEX	<0.001	<0.001	-2.39	RPS3, RPS8, RPS24, RPS17, RPS3A, EIF3E, RPS4X, RPS20, RPS6, RPS5, RPS18, EIF3D, RPS21, EIF3C, RPS23, RPS11, RPS2, RPS12, EIF3H, EIF3B, RPS27A, RPS29, RPS14, RPS9, EIF1AX, RPSA, EIF3J, RPS4Y1, RPS10, EIF3F, RPS7, RPS15, RPS25, RPS27, RPSAP9
YAGI_AML_FAB_MARKERS	<0.001	<0.001	-2.37	MYO16, GABRE, DLC1, TRPC6, PLOD2, RYR3, LAPTM4B, FGFR3, GAD1, GATA2, ITGA2B, ADAMTS3, PBX1, RHAG, CXADR, KEL, CFH, LAMA5, PROS1, MAST4, CDC42BPA, SERPINI1, TOM1L1,

				HERC2P2, PIR, TIMP3, PKIG, NET1, TRPC1, TRIB2, ULK2, SERPINE2, MYH10, PKIA, ALDH5A1, CLCN4, ZBTB16, SNTA1, LDB1, BAMBI, SERPINH1, GP1BA, UBXN8, ZMYND8, MYL4, ELOVL6, MLLT11, TGM2, CSF2RB, TPM1, PLA2G6, TFDP2, PCCB, FADS1, DDHD2, ACO1, CTSW, HDGFRP3, MORF4L2, FAM20B, PAFAH1B3, PDCD10, TM7SF2, TGIF1, ICAM2, BAG2, CAV1, DRAP1
REACTOME_TRANSLATION	<0.001	<0.001	-2.33	RPL36A, RPL31, RPS3, RPS8, EIF4EBP1, RPS24, RPL9, RPS17, RPS3A, EEF1G, RPL12, EIF2B4, EEF2, EIF3E, RPS4X, RPL7, RPL36, RPS20, RPS6, RPS5, RPL3, RPL10A, RPS18, EEF1B2, RPLP0, EIF3D, RPS21, RPL15, EIF3C, RPL29, RPS23, RPL24, RPL37A, RPL14, RPL7A, RPL5, RPS11, RPS26, RPL8, RPL32, RPS2, RPS12, RPL4, EIF3H, RPL41, EIF3B, RPL22, RPL27A,

				RPS27A, RPL18A, RPS29, EIF4G1, RPL35, RPL13A, RPL37, RPL10, RPS14, RPS9, RPL18, EIF1AX, RPSA, EIF3J, RPS4Y1, SRP14, RPL30, RPS10, RPL13, EIF3F, RPS7, RPS15, RPL23, RPS25, RPS27, RPL19, EIF4A1, RPL26, EEF1A1, RPL23A, RPSAP9, RPL34, EIF4E, SRPRB, RPL11, RPL35A, EIF5B, ETF1, RPL28, RPL17, DDOST, RPS19
HALLMARK_MYC_TARGETS_V1	<0.001	<0.001	-2.27	MYC, IMPDH2, APEX1, NPM1, CCT2, RPS3, HSP90AB1, NME1, TRIM28, CDK4, POLD2, GNB2L1, HNRNPA1, SMARCC1, GNL3, NOLC1, RPS6, RPS5, C1QBP, ILF2, CCT3, NOP56, HDAC2, TCP1, EEF1B2, RPLP0, EIF3D, PHB, SET, SYNCRIP, CCT5, PABPC4, IARS, ACP1, PA2G4, HSPD1, CCT4, RPL14, RUVBL2, SNRPD1, CAD, LSM7, CCT7, YWHAE, NHP2, NOP16, NCBP2, G3BP1, CSTF2,

				MCM7, SSBP1, AIMP2, PHB2, RPS2, CLNS1A, FBL, ODC1, CDK2, PSMD3, EIF3B, GLO1, RPL22, RAN, SNRPA, LSM2, KPNB1, RSL1D1, TUFM, SNRPD2, RPL18, GSPT1, UBE2E1, EIF1AX, EIF3J, MRPS18B, CYC1, SRSF2, GOT2, TOMM70A, RPS10, SERBP1, SLC25A3, YWHAQ, HDDC2, PRDX4, HNRNPR, MRPL9, PTGES3, MCM2, RANBP1, COPS5, PSMA1, EIF4A1, KARS, ABCE1, RPL34, IFRD1, DDX21, EIF4E, SRSF1, VDAC1, SF3B3, CBX3, SRSF7, SRM, SNRPA1, PWP1, ETF1, SRSF3, DDX18, RNPS1, ORC2, RRP9, PSMA6, EIF4G2, RPL6, SSB, HNRNPC, XPOT, EPRS, PSMD8, TRA2B, MCM4, PPIA, PSMD14, EXOSC7
SCHURINGA_STAT5A_TARGETS_	<0.001	0.001	-2.22	RHAG, ANK1, GYPB, CD36, XK, RAC3, LEPR, MAOA, PIM1, HBE1, TRAF4, CISH

UP				
KEGG_STEROID_HORMONE_BIOSYNTHESIS	0.003	0.015	-1.97	CYP7B1, AKR1C1, AKR1C2, AKR1C3, HSD17B8
SCHUHMACHER_MYC_TARGETS_UP	<0.001	0.024	-1.95	MYC, MEST, AK4, PYCR1, IMPDH2, SORD, TARBP1, TMEM97, SLC39A14, FABP5, EBNA1BP2, NME1, FXN, CDK4, RABEPK, POLD2, TFRC, SLC16A1, NOLC1, UCK2, DDX10, TBL3, AHCY, GCSH, PAICS, IARS, PPAT, FKBP4, PEBP1, CAD, DHODH, GRSF1, AIMP2, RRS1, POLR2H, ODC1, FASN, ZNF239, PRDX4, EXOSC2, RANBP1, MRPL3, AUH, ABCE1, DDX21, KIAA0020, RCC1, SRM, BOP1, CTSC, MTHFD1
REACTOME_METABOLISM_OF_R	<0.001	0.032	-1.9	RPL36A, EXOSC4, RPL31, RPS3, RPS8, RPS24, MAPK11, RPL9, RPS17, PRMT5, RPS3A, ZFP36L1,

NA				PSMB5, RPL12, CNOT7, NUP43, RPS4X, RPL7, RPL36, TNKS1BP1, RPS20, PAIP1, RPS6, RPS5, GEMIN6, RPL3, RPL10A, RPS18, RPLP0, PSMD6, RPS21, RPL15, NUP35, RPL29, EXOSC6, SEH1L, RPS23, LSM1, RPL24, RPL37A, RPL14, SNUPN, RPL7A, AAAS, RPL5, SNRPD1, RPS11, RPS26, NCBP2, RPL8, RPL32, PSMC2, KHSRP, RPS2, CLNS1A, RPS12, RPL4, PSMD3, RPL41, SNRPE, RPL22, RPL27A, RPS27A, RPL18A, RPS29, EIF4G1, PPP2R2A, RPL35, LSM2, RPL13A, RPL37, RPL10, RPS14, SNRPD2, RPS9, RPL18, UPF3A, RPSA, GEMIN2, NUP153, RPS4Y1, RPL30, RPS10, GEMIN5, SNRPF, RPL13, RQCD1, DCP1B, CNOT10, SMG9, RPS7, RPS15, RPL23, EXOSC2, SMN1, TGS1, RPS25, RPS27, RPL19, PSMA1, EIF4A1, AKT1, NUP62, RPL26,

				RPL23A, GEMIN4, NUP93, RPSAP9, RPL34, LSM5, PSMB6, EIF4E, SNRPB, RPL11, RPL35A, PPP2R1A, ETF1, PSMB7, RPL28, RNPS1, RPL17, UPF3B, HSPB1, RPS19, PSMA6, PHAX, CNOT8, PSME4, RPL6, EIF4B, DDX20, PSMD4, EXOSC3, EXOSC8, PSMD10, PSMD8, EIF4A3, WDR77
HALLMARK_MYC_TARGETS_V2	<0.001	0.004	-1.87	MYC, SORD, TMEM97, NPM1, DCTPP1, RCL1, CDK4, PPAN, RABEPK, BYSL, GNL3, NOLC1, NOP56, NDUFAF4, PHB, PES1, UNG, MRTO4, PA2G4, HSPD1, WDR74, FARSA, PUS1, TBRG4, PRMT3, NOP16, IPO4, AIMP2, WDR43, TCOF1, IMP4, GRWD1, MYBBP1A, SLC29A2, PPRC1, TFB2M, NOP2, CBX3, SRM, DDX18, RRP9
REACTOME_FORMAT	0.009	0.046	-1.83	TUBB4A, TUBB6, CCT2, TUBA1A, TUBA1C, TUBB2B, CCT3, TCP1,

ION_OF_TUBULIN_FOLDING_INTERMEDIATES_BY_CCT_TRIC				TUBB2A, CCT5, CCT4, CCT7, CCT8, CCT6A, TUBB3
REACTOME_PLATELET_AGGREGATION_PLUG_FORMATION	0.004	0.123	-1.67	MPL, GP1BB, ADRA2A, ITGA2B, RAPGEF4, GP1BA, PTK2
PID_MYC_ACTIVPATHWAY	<0.001	0.126	-1.66	MYCT1, SERPINI1, MYC, BCAT1, FOSL1, PIM1, POLR3D, NPM1 NME1, CDK4, PDCD10, TP53, TFRC, CCND2, HMGA1, MINA, TAF9, SMAD3, NME2, MTA1, MAX, KAT2A, HSPD1, RUVBL1, BMI1, RUVBL2, TAF4B, CAD, SNAI1, ODC1, SLC2A1, EIF4G1, NBN, ACTL6A, EIF4A1, HSPA4, PMAIP1, EIF4E, PTMA, NCL, RCC1, RPL11,

				HSP90AA1, DDX18
HALLMARK_HEME_METABOLISM	<0.001	0.135	-1.42	RHAG, KEL, MINPP1, ANK1, GYPB, SLC7A11, SELENBP1, GYPE, XK, TAL1, CLIC2, GATA1, BMP2K, NFE2, ICAM4, SPTA1, KLF1, RAP1GAP, HBD, BCAM, ADD2, MYL4, TRIM58, MBOAT2, SMOX, ABCB6, CA1, MOCOS, FECH, FN3K, TFDP2, UBAC1, MPP1, RCL1, HBQ1
HALLMARK_FATTY_ACID_METABOLISM	0.018	0.16	-1.35	ACSM3, AADAT, CD36, CEL, BPHL, LTC4S, APEX1, MAOA, ACOT2, MIF, NTHL1, REEP6, CPT1A, EHHADH, GSTZ1, BCKDHB, ALDH3A1, PCBD1, CA2, UROD, ALDH1A1, GRHPR, UGDH, ADSL, CRAT, PPARA, HIBCH, DECR1, ACAA2, ODC1, EPHX1, ECI2, FASN, PRDX6, ELOVL5, HADH, ALAD, SMS, PTPRG, GLUL, NBN, HMGCL, SETD8, ADIPOR2, GCDH, OSTC, AUH, ECHS1, DLST,

				ACADM, ACADS, HSP90AA1, ECH1, GPD2, HMGCS1, CPT2

enriched gene sets in BMMC of Case 1 in relapsed status versus after ADI-PEG20 in complete remission status

Gene set	nominal p-value	FDR q-value	NES*	Core genes
ROSS_AML_OF_FAB_M7_TYPE	<0.001	<0.001	3.01	DLC1, LAPTM4B, RYR3, ITGA2B, PLOD2, RHAG, TFR2, GATA2, APOE, PROS1, STXBP6, MINPP1, SERPINI1, PNMT, KEL, APOC1, NEO1, TIMP3
YAGI_AML_FAB_MARKERS	<0.001	<0.001	2.5	MYO16, GABRE, DLC1, ADAMTS3, TOM1L1, LAPTM4B, RYR3, ITGA2B, PLOD2, FGFR3, GAD1, RHAG, CXADR, GATA2, PROS1, CFH, CDC42BPA, PBX1, SERPINI1, KEL, TRPC1, TRIB2, HERC2P2, PIR, UBXN8, TIMP3, NET1, TUSC3, SERPINE2, TRPC6, ALDH5A1, CLCN4, PKIG, MYH10, PLA2G6, ELOVL6, MLLT11, PKIA, PCCB, LDB1, PAQR3, LAMA5, BAG2,

				ULK2, NLRP3, CD200, CSF2RB, ILF2, ALDH1A1, MORF4L2, PDCD10, ACO1, TGM2, NAE1, SERPINH1, ZMYND8, CTSW, FAM20B, RFC3, FADS1
HALLMARK_MYC_TARGETS_V1	<0.001	<0.001	2.19	MYC, IMPDH2, NPM1, C1QBP, CCT2, APEX1, HSPD1, MAD2L1, EEF1B2, GLO1, SNRPD1, SNRPG, CLNS1A, NME1, CDK4, HNRNPA1, PSMC6, ILF2, HSP90AB1, NDUFAB1, ERH, GNL3, TCP1, ABCE1, TOMM70A, CCT4, VBP1, MRPL9, CCT3, IARS, RSL1D1, EIF4E, AIMP2, CCT5, HDAC2, KPNA2, PSMA2, RPL14, DDX18, RPL34, YWHAQ, EIF3D, EIF1AX, NCBP2, MRPS18B, PSMD14, PSMD8, PWP1, DUT, EPRS, PSMA6, G3BP1, EIF2S1, PPIA, UBE2E1, SRSF1, RAN, SNRPB2, SNRPD2, PHB, LSM7, PRDX3, KARS, UBA2, SET, VDAC3, RPS5, PSMA4, NOLC1, PA2G4, MCM4, SRSF7,

				SSBP1, RPL22, CYL1, RPLP0, SMARCC1, GNB2L1, CCNA2, XPOT, CBX3, HSPE1, COPS5, RUVBL2, PPM1G, HDDC2, SERBP1, AP3S1, XRCC6, CDK2, MCM2, POLD2, NOP56, PHB2, SSB, PCNA, DHX15, GSPT1, RRM1, VDAC1, PSMD7, SNRPD3, EIF3B, PRDX4, SLC25A3, PSMA7, CNBP, RPS6, ACP1, DDX21, CANX, SF3B3, EIF2S2, XPO1, ORC2, RFC4, PABPC4, YWHAE, EIF4A1, POLE3, HNRNPC, BUB3, HNRNPR, PSMC4, TRA2B, SRSF2, PSMA1, MCM7, ODC1, STARD7, PSMD1, PSMD3, SRSF3, RAD23B, COX5A, DEK, ETF1, H2AFZ
SEKI_INFLAMMATORY_RESPONSE_LPS_UP	<0.001	0.013	2.04	FAM84A, CCL3, CXCL2, IER3, ADAMTS7, CXCL10, EXOC3L4, CSF1, NFKBIZ, NFKBIA, EREG, MAFF, TSLP, CCRL2, RIPK2, SOD2, GBP1

WONG_MITOCHONDRIA_GENE_MODULE	<0.001	0.017	2	SLC9A2, SLC40A1, MAOA, TRIM45, MRPS33, GCAT, NT5M, MRPL36, MRPL13, COQ3, PCCB, ABCB6, MRPL42, MRPL45, ACAT1, AGMAT, UNG, NDUFS4, SOD2, HSCB, DCTN6, NDUFB4, NDUFAF1, NDUFAB1, MTHFD2, NDUFB1, TOMM70A, ALDH6A1, COX6C, PDHX, MRPL40, PDHB, MRPS30, MRPS36, DECR1, TFAM, UQCRB, MRPS18B, ATP5F1, FH, NIPSNAP1, MTRR, MCCC2, NDUFA7, DLD, DUT, SLC25A32, UQCRQ, SCO1, MRPS17, NME4, MRPL32, KARS, CRYZ, VDAC3, COX7B, MRPL3, ATP5C1, NDUFV2, MRPS28, ATOX1, CYC1, PDK3, ATP5J, CRYZL1, TIMM10, NQO1, MRPL18, NDUFA4, TIMM23, TIMM13, NDUFS3, SEC61G, CPT1A, WARS2, TOMM7, NDUFA5, NDUFA12, COX18, NDUFB6, ECHS1, NDUFA8, MRPL11, MCAT,

				ATP6V1E1, ATP5G2, GPX4, COX7A2, COX7C, DAP3, NDUFB3, UQCRFS1, ATP6V1C1, ATP5B, ATP5O, ATP6V1G1, MRPL49, NDUFS8, NDUFB8, NDUFB10, NDUFA6, ATP5I, MRPS15, NDUFB5, NDUFS5, MRPL27, ATP5L, NDUFV1, UQCR10, HSD17B10, PTRH2, VDAC2, COX5A, COX6A1, ATP13A3, AUH, COX7A2L, ATP6V1D, UQCR11, IDH2, SDHB
VANASSE_BCL2_TARGETS_UP	<0.001	0.044	1.85	RELN, RYR2, SLC16A9, MACC1, SERPING1, LIFR
HALLMARK_TNFA_SIGNALING_VIA_NFKB	<0.001	0.041	1.66	IL1B, SERPINE1, CXCL2, CXCL3, IER3, CXCL10, EDN1, CSF1, F3, MYC, CCL4, PTGS2, LAMB3, NFKBIA, BCL2A1, DNAJB4, DUSP1, ETS2, G0S2, MAFF, PDLIM5, ZBTB10, TNFAIP6, BTG3, CCRL2, YRDC, RIPK2, KYNU, IL18, SOD2, CXCL11, TNF, CD83,

				STAT5A, PTGER4, SERPINB2, CD69, EGR1, KLF9, ATF3, NAMPT, SGK1, MSC, TRIP10, MARCKS, CCL2
HALLMARK_MYC_TARGETS_V2	0.012	0.024	1.62	MYC, NDUFAF4, SORD, NPM1, HSPD1, TMEM97, RABEPK, UNG, CDK4, WDR74, GNL3, AIMP2, IMP4, NIP7, DDX18, FARSA, MPHOSPH10, PHB, RCL1, WDR43, NOLC1, PA2G4, MCM4, MRTO4, CBX3, HSPE1, DCTPP1
HALLMARK_INFLAMMATORY_RESPONSE	<0.001	0.031	1.56	IL1B, SERPINE1, SLC1A2, KCNJ2, DCBLD2, CXCL9, CXCL10, EDN1, CSF1, F3, MYC, ADORA2B, MMP14, ICAM4, NFKBIA, EREG, ITGB8, FFAR2, CALCRL, TNFAIP6, AQP9, CCRL2, MSR1, TLR3, RIPK2, TIMP1, NLRP3, IL18, AHR, RNF144B, ATP2C1, CXCL11, IL10, LAMP3, NM1, PTGER4
HALLMARK_FATTY_	0.005	0.033	1.54	AADAT, BMPR1B, CEL, MAOA, LTC4S, ACSM3, CD36, G0S2, OSTC,

ACID_METABOLISM				MIF, APEX1, CCDC58, BPHL, PTPRG, UROD, ACOT2, INMT, NBN, NTHL1, EHHADH, ALDH1A1, CA2, HIBCH, PCBD1, PTS, PDHB, DECR1, MDH1, ECI2, FH, ADSL, HMGCS1, DLD, SDHD, HPGD, GRHPR, SMS, CPT2, ACAA2, CRYZ, ACADM, HADHB
HALLMARK_E2F_TARGETS	0.005	0.072	1.44	DSCC1, MYC, LYAR, GINS1, CKS2, WEE1, MAD2L1, PAICS, UNG, NBN, TFRC, NME1, CDK4, HMGB3, CDKN2A, ASF1A, MTHFD2, EXOSC8, NUDT21, RFC3, NUP107, KPNA2, ORC6, HN1, DCK, DUT, POP7, UBE2T, EIF2S1, RFC2, SRSF1, RAN, ZW10, DLGAP5, PPP1R8, CSE1L, CCNB2, DEPDC1, NOLC1, CKS1B, PA2G4, MCM4, NUP205, RAD21, MCM3, DCTPP1, RACGAP1, XRCC6, MCM2, ING3, POLD2, PRKDC, NAA38, NOP56, RPA2, PCNA, STMN1, GSPT1, BUB1B, RPA3, PHF5A, CDK1, SLBP,

				HMMR, PRDX4, SMC6, UBE2S, CDKN3, KIF2C, PLK4, HMGA1, XPO1, RQCD1, BRCA2, ORC2, PTTG1, CDKN2C, IPO7, TRA2B, ANP32E, SRSF2, PNN, MCM7, SSRP1, RAD51AP1, NUP153, RBBP7, TOP2A, CHEK1, MLH1, DEK, H2AFZ, SMC4, TP53, AURKA
HALLMARK_IL6_JAK_STAT3_SIGNALING	0.031	0.075	1.42	IL1B, CXCL3, CXCL9, CXCL10, CSF1, IL7, PDGFC, CD36, LEPR, TNFRSF12A, IL1R2, PIM1, SOCS1, CSF2RB, CD38, CXCL11, A2M, TNF
HALLMARK_HEME_METABOLISM	0.005	0.088	1.39	RHAG, MINPP1, KEL, CLIC2, XK, SELENBP1, SLC7A11, ANK1, ICAM4, TAL1, KLF1, SPTA1, BMP2K, NFE2, CA1, MBOAT2, ABCB6, FECH, BCAM, UROD, GATA1, TFRC, GYPA, RAP1GAP, TSPO2, GMPS, CA2, MPP1, RHCE, ALDH6A1, ABCG2, HBD, TCEA1, GCLC, TMCC2, GYPB, TRIM58, LMO2, UBAC1, CAT, TRAK2, RCL1,

				BPGM, SMO
HALLMARK_XENOBIOTIC_METABOLISM	0.049	0.158	1.3	SERPINE1, GAD1, PROS1, AKR1C2, MAOA, G6PC, BCAT1, CASP6, ACOX2, CD36, ETS2, CROT, PDLIM5, AQP9, BPHL, TMEM97, KYNU, AKR1C3, RAP1GAP, ADH5, PYCR1, TPST1, CA2, ENPEP, GSS, JUP, PTS, GCLC

enriched gene sets in BMMC of Case 2 before ADI-PEG20 versus after ADI-PEG20

Gene set	nominal p-value	FDR q-value	NES*	Core genes
PID_SYNDECAN_1_PATHWAY	0.013	0.141	-1.74	COL4A1, COL16A1, COL5A2, COL8A1, COL12A1, COL14A1, COL1A2, COL3A1, MET, COL6A3, COL5A1, COL7A1, COL4A4, COL8A2
NABA_COLLAGENS	0.011	0.154	-1.73	COL4A1, COL16A1, COL5A2, COL8A1, COL12A1, COL14A1, COL6A5, COL1A2, COL6A6, COL28A1, COL3A1, COL4A2, COL6A3, COL5A1, COL7A1,

				COL4A4, COL8A2, COL19A1, COL4A3, COL26A1, COL24A1
VALK_AML_CLUSTER_8	0.015	0.267	-1.73	GYPA, RHCE, RHD, GYPB, SNCA, ABCG2, TNS1, TAL1, CDH1, ANK1, TSPAN5, PBX1, TRAK2, SELENBP1, ARHGEF12
NAKAYAMA_FGF2_TARGETS	0.021	0.362	-1.69	COL4A1, GPT, COL5A2, COL8A1, IGF1, OLFML3, CXCL12, PDGFRB, MSMO1
REACTOME_COLLAGEN_FORMATION	0.021	0.265	-1.65	COL4A1, COL16A1, COL5A2, ADAMTS2, COL8A1, COL12A1, COL14A1, COL1A2, COL28A1, COL3A1, ADAMTS3, COL4A2, TLL1, COL6A3, COL5A1, COL7A1, PCOLCE, COL4A4, PLOD2, COL8A2, COL19A1, LEPREL1, SERPINH1
HALLMARK_HEME_METABOLISM	0.015	0.346	-1.35	CTSE, GYPE, GYPA, RHCE, RHD, GYPB, TSPO2, ACSL6, CA1, AHSP, RHAG, KEL, SNCA, ABCG2, EPB42, TNS1, SPTB, SPTA1, SLC6A9, TAL1, RAP1GAP, MINPP1, ADD2, CPOX,

				TRIM58, PDZK1IP1, ANK1, CA2, ABCB6, HBB, TMCC2, TSPAN5, IGSF3, SLC2A1, HMBS, HBQ1, HBD, TRAK2, SELENBP1, PC, ERMAP, UROD, TFRC, SLC25A37, ARHGEF12, BPGM, ENDOD1, PPOX, HBBP1, DAAM1, PIGQ, ISCA1, SLC11A2, GLRX5, XPO7, HEBP1, GCLC, SLC30A1, ALAD, FAM46C, MARCH8, AGPAT4, EIF2AK1, MFHAS1, EPB41, AQP3, RNF123, RANBP10, BMP2K, E2F2, HBZ, CLCN3, RCL1, HIST1H4C, MGST3, BTRC, BNIP3L, UCP2, ASNS, UBAC1, GYPC, DCUN1D1, CROCCP2, KAT2B, UROS, NUDT4, NFE2, MXI1, NCOA4, OPTN, BTG2, SLC7A11, SLC25A38, HDGF, TCEA1, CAT, CTNS, ALDH6A1, BLVRA, KLF3

*NES: Normalized enrichment score

Supplementary Table 3.

enriched gene sets in BMMC of Case 1 and Case 2 before ADI-PEG20 (BMMC1B and BMMC2B) versus 3 non-responders

Gene set	nominal p-value	FDR q-value	NES*	Core genes
ROSS_AML_OF_FAB_M7_TYPE	<0.001	<0.001	2.68	RYR3, RHAG, PCDH9, ANK1, KEL, TFR2, ITGA2B, TAL1, KCNH2, NEO1, PROS1, DLC1, ALDH1A1, MYH10, GP1BA, DNAJC6, KLF1, GATA1, MINPP1, SERPINI1, PNMT, FHL2, APOC1, DNM3, TPM1, PDLIM5, UROD, SDPR, PLOD2, TIMP3, BMP2K, STXBP6, CMAS, ABCC4
YAGI_AML_FAB_MARKERS	<0.001	0.01	1.98	RYR3, RHAG, ADAMTS3, PBX1, KEL, GAD1, PIR, ITGA2B, PROS1, DLC1, CXADR, ALDH1A1, LAMA5, MYH10, GP1BA, SERPINI1, CLCN4, PKIA, CDC42BPA, GP9, TPM1, CAV1, TRIB2, SNTA1, ULK2, TRPC1, PLOD2, ELOVL6,

				HDGFRP3, NTRK1, CPM, TIMP3, PKIG, CACNA2D2, NR4A2, TGM2, CFH, NCAM1, LAPTM4B, NET1, HERC2P2, CSF2RB, UBXN8, MAST4, RRAS2, LDB1, GATA2, ATXN1, ELL2, SERPINE2, STAT4, FGFR3, TBPL1, RFC3, PDCD10, ANXA5, MED21, F2R, CAST, CD164, DLEU1, ELOVL5, NAE1
OLSSON_E2F3_TARGETS_UP	0.004	0.029	1.86	PTGER3, C1orf198, ELF3, AKR1C2, DAG1, ECE1, PVRL1
SCHURINGA_STAT5A_TARGETS_UP	0.007	0.055	1.76	RHAG, ANK1, XK, CD36, LEPR, VEGFA
REACTOME_PLATELET_AGGREGATION_PLUG_FORMATION	0.006	0.118	1.72	GP1BB, ITGA2B, PTK2, GP1BA, GP9, ADRA2A, GP5, FN1, VWF, RASGRP1
PID_WNT_NONCANONICAL_PATHWAY	0.013	0.211	1.63	ROR2, MAPK10, FZD6, DAAM1, FZD5, FZD7

HALLMARK_TNFA_SIGNALING_VIA_NFKB	0.013	0.12	1.36	G0S2, CCND1, LAMB3, BMP2, EGR2, TUBB2A, CSF1, CYR61, EGR3, PDLIM5, PPAP2B, PPAP2B, CXCL10, FOSL1, INHBA, CD80, GPR183, TNFRSF9, EGR1, CD83, BIRC3, MARCKS, NR4A2, NR4A1, CLCF1, KYNU, DUSP4, TRIP10, MAFF, SMAD3, VEGFA, BTG1, PTGS2, TNFAIP6, SERPINE1, IFIT2, SLC2A3, PANX1, SOD2, BTG2, RHOB, DUSP2, NAMPT, DNAJB4, CD69, HBEGF, SGK1, IRS2, ID2, YRDC, NR4A3, IL1B

enriched gene sets in pre-ADI-PEG20 BMMC of 3 non-responders versus 2 responders

Gene set	nominal p-value	FDR q-value	NES*	core genes
SCHURINGA_STAT5A_	<0.001	0.019	-1.96	MPO. CEBPA, ELANE, CTSG, TYROBP, CD44, PECAM1

TARGETS_DN				
VALK_AML_CLUSTER_10	<0.001	0.019	-1.93	AZU1, MN1, CFD, CHRDL1, SETBP1, LPHN1, RNASE2, RBPMS, SPON1, PPP1R16B, NPDC1, SPTBN1, FLNB, F2RL1, CRIM1, PRKD2, CD22
KAMIKUBO_MYELOID_CEBPA_NETWORK	<0.001	0.018	-1.92	PRTN3, CEBPA, EMR1, RAB31, LGALS1, CSF2RA, HP, CYBB, IL18
VILIMAS_NOTCH1_TARGETS_DN	0.011	0.123	-1.73	MPO, VPREB1, CTSG, CSF2RA, LRRC25, GFI1, CSF3R
CREIGHTON_AKT1_SIGNALING_VIA_MTOR_UP	0.004	0.135	-1.7	ARHGEF16, BIK, CYB561, CLDN3, SLC37A1, DDR1, DUSP10, NEU1, TJP3, PRKCD, UBE2M, LASP1, CLSTN1

VALK_AML_WITH_FLT3_ITD	0.015	0.18	-1.66	HOXB6, HOXA9, HOXB3, APP, BAHCC1, IL2RA, MMP2, LCT, LGALS3BP, QPRT, TRPC2, MAP1A, TRIM16, GOLGA8A, IL1RAP, PBX3
WEIGEL_OXIDATIVE_STRESS_RESPONSE	0.019	0.228	-1.61	CDH3, APP, COMT, CASK, POLD1, BAX, IGFBP2, ARHGDIA, ZNF91, NF2, XIAP, DAPK1, ITGA5, CUX1, GNAS, CAPNS1
GENTLES_LEUKEMIC_STEM_CELL_DN	0.03	0.226	-1.6	CCNA1, STAR, RNASE3, RNASE2, MS4A3, CSTA

NES*, Normalized enrichment score

Supplementary Figure 1. Potentially deleterious genetic mutations in Case 1 and Case 2

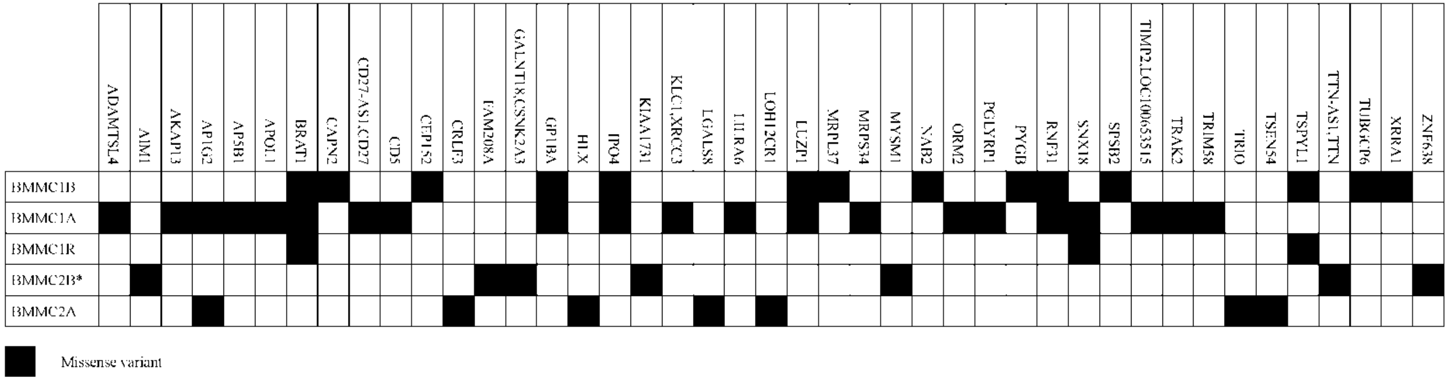

*, Mutations with change of protein coding in BMMC2B

BMMC1B and BMMC2B are BMMC in case 1 and case 2 before ADI-PEG20.

BMMC1A and BMMC2A are BMMC in case 1 and case 2 after ADI-PEG20 in complete remission status

BMMC1R is BMMC in case 1 in relapsed status.
